# Viral Chemokine Receptors

**DOI:** 10.3389/fimmu.2015.00281

**Published:** 2015-06-05

**Authors:** Philip M. Murphy

**Affiliations:** ^1^Molecular Signaling Section, Laboratory of Molecular Immunology, National Institute of Allergy and Infectious Diseases, National Institutes of Health, Bethesda, MD, USA

**Keywords:** herpesvirus, chemokine, receptor, HIV, AIDS

When my lab sequenced the first chemokine receptors CXCR2 and CCR1 in 1991, the top BLAST hit for CCR1 was open reading frame (ORF) *US28* of human cytomegalovirus (HCMV), indicating an obvious common ancestor and a possible example of gene piracy. Pox virologists had already identified virally encoded TNF and IFN-γ binding proteins, copied from the host and redeployed as cytokine scavengers and immune evasion factors; however, there were no precedents for G protein-coupled receptors in viruses or signaling viral immunoreceptor homologs. Tom Schall, then at Genentech, who had candidate CC chemokine receptor clones, learned about our CCR1 discovery and proposed a collaboration. I told him about the key features of CCR1: its specificity for CCL3 and CCL5, as well as monocytes and lymphocytes, and its sequence homology to US28 (1); however, he eventually wanted to pursue CCR1 independently, and published it in *Cell* in fall, 1992 along with data that US28 could bind to CCL2, CCL3, CCL4, and CCL5. A month earlier, our paper had been rejected by *Cell* for lack of sufficient binding data; however, we eventually published our paper in early 1993 in the *Journal of Experimental Medicine* (1), and the NIH received the patent for cloning CCR1.

Meanwhile, the complete sequence of the non-human primate herpesvirus *Herpesvirus saimiri* appeared in 1992, revealing a CXCR2 homolog known as ECRF3. Sunil Ahuja, then a post-doc in my lab and now a Professor at the University of Texas at San Antonio, used our *Xenopus* oocyte expression system to show that ECRF3, like CXCR2, mobilized calcium in response to the ELR^+^ CXC chemokines CXCL1, CXCL7, and CXCL8, the first example of a virally encoded chemokine receptor that signaled (2). My long-time colleague Ji-Liang Gao, who was a post-doc in my lab at the time, then showed that US28 was also a calcium flux signaling receptor for the same chemokines that Schall’s group had found bound to US28 (3). Together these papers pioneered a new field of virally encoded chemokine receptors that has expanded as more herpesvirus and poxvirus genomes have been sequenced. In addition, many virally encoded chemokines and secreted chemokine binding proteins were later identified, along with information about structure, signaling pathways, biological functions, and potential disease connections.

Together, this work demonstrated unequivocally that the chemokine system has been selectively and preferentially expropriated by these types of viruses; however, exactly why remains unresolved. At an evolutionary level, it is remarkable that the viral chemokine receptors could be so distantly related to mammalian receptors with which they share ligands, particularly since this was not the case for the human chemokine receptors known at the time, CXCR1, CXCR2, CCR1, and CCR2.

We also began cloning mouse counterparts of the human receptors we were finding and noticed that the mouse–human orthologs were more distantly related than expected. I decided to investigate this systematically by doing an *in silico* study of the mouse–human orthologs then in the data base, and found that for the ~500 available sequence pairs, the distribution of divergence was highly heterogenous. Most orthologs had high homology, but the ones that did not were mostly immunoregulatory factors. I published a paper in *Cell* describing this exceptionalism and proposed that it might relate to evolutionary pressure imposed by the predilection of viruses for this type of host gene (4).

We continued to use homology cross-hybridization to clone additional chemokine receptors, including, in 1994, one we first named CC CKR5 that was later renamed CCR5. Christophe Combadiere, a post-doc in my lab now with his own lab in Paris, actually cloned CCR3 and CCR5 cDNAs from the same screen. He determined their sequences and leukocyte specificities in parallel, then investigated their chemokine specificities sequentially, starting with the eosinophil-selective CCR3. We reported that CCL3, CCL4, and CCL5 were agonists for CCR3 in May, 1995, accepted by *JBC* 1 day after submission with no revisions (5). However, colleagues in the field, and ultimately we were skeptical since CCL3 and CCL4 lacked eosinophil activity. As a check, Christophe sequenced transfected cDNA from the original “CCR3” cell lines and, to our chagrin, found the CCR5 sequence, not CCR3. Apparently, the plasmid tube labeled “CCR5” had been mistaken for “CCR3”. We wrote a correction published in December, 1995 in *JBC* indicating that CCL3, CCL4, and CCL5 were agonists for a new receptor named CC CKR5. We then submitted a new paper with the CC CKR5 sequence, its RNA distribution, and ligands to *JBC*, which after an ~6 month review was rejected in part because the reviewers regarded it as partly duplicative of the CCR3 paper.

The same month that our correction appeared in *JBC*, Paolo Lusso and colleagues in Bob Gallo’s lab at the NCI of NIH reported in *Science* that CCL3, CCL4, and CCL5, the signature ligands for CCR5, were able to suppress replication of macrophage (M)-tropic but not T cell line (T)-tropic strains of HIV (6). Taken together, the most obvious and parsimonious hypothesis was that CCR5 was used for M-tropic HIV infection, but how was unclear to me. On January 31, 1996, I gave a seminar at NIH about our new chemokine receptors, concluding with Paolo’s new finding about HIV-suppressing chemokines and how CCR5 was an ideal candidate to mediate their action. My colleague Ed Berger from NIAID and his staff were at the talk, and Ed emailed me a few days later about his recent unpublished work identifying fusin (later identified as a chemokine receptor for CXCL12 and renamed CXCR4) as the first HIV coreceptor, acting with CD4 at the level of cell entry, and its specificity for T-tropic strains of HIV (7). He said he was still looking for a specific HIV coreceptor for the disease-transmitting M-tropic HIV and agreed that CCR5 was the logical candidate. This is how I realized that CCR5 might work at the level of cell entry, from Ed, and of course his lab had the assay to test the idea. From me, he learned about the CCR5 sequence and its leukocyte and chemokine specificities that matched Paolo’s chemokine suppressor signature. We provided the plasmid to Ghalib Alkhatib, a post-doc in Ed’s lab, who validated the hypothesis on the first attempt and highlighted the result by writing “BINGO!” in his lab notebook.

Meanwhile, Marc Parmentier from Brussels had beaten us to press with his own independent cloning and functional characterization of CCR5, in *Biochemistry* on March 19, 1996 (8). This was the key piece needed to allow four other labs to join the CCR5-HIV connection frenzy that year, that started with Paulo’s *Science* paper about three CC chemokine suppressors of M-tropic HIV, our *JBC* correction reassigning these same chemokines from CCR3 to the unpublished CCR5 sequence, followed in February, 1996 by Ed’s pre-publication announcement of fusin’s T-tropic HIV coreceptor activity at a Keystone meeting. To the astute observer, the only missing piece to the puzzle was the CCR5 sequence, provided first by Parmentier. Within 2 weeks of each other in late June, 1996, all five groups in the hunt published papers in *Science*, *Nature*, and *Cell* that used complementary approaches to draw the same basic conclusion that CCR5 was an M-tropic HIV coreceptor (9, 10). Two weeks after that, we published the sequence of CCR5 with its leukocyte and chemokine specificities, at last, in the *Journal of Leukocyte Biology* (11). The foundational discovery, the first HIV coreceptor fusin/CXCR4, was published by the Berger lab 1 month earlier in *Science* (7). The pace of discovery and publication had become breathlessly exciting, and the pages of scientific journals as well as the lay press were ablaze with stories of the HIV-chemokine receptor connection, for the new insights as well as for the potential for new drugs targeting a host factor in HIV/AIDS.

Ironically, Ed and I had first met several years earlier when he came to my lab to ask about using my oocyte system to expression clone a putative HIV coreceptor from his cDNA library. We never actually did any experiments then, and instead ended up with the converse collaboration: using my lab’s CCR5 cDNA clone in Ed’s system to identify the M-tropic HIV coreceptor. Ultimately, my lab’s contribution was to accelerate the discovery of CCR5 as the M-tropic HIV coreceptor, since Ed’s expression cloning system used to find fusin/CXCR4 would probably have succeeded in also discovering CCR5.

But what role did these coreceptors actually play in pathogenesis? My lab took a lead in answering this next key question, through the discovery of *CCR5Δ32*, the deletion mutant of *CCR5*, which provided strong evidence that CCR5 was critical for HIV transmission at the population level. I thought that if a common inactivating CCR5 mutation existed, homozygotes should be rare among HIV-infected individuals, but overrepresented among highly exposed but persistently uninfected individuals. I proposed the idea of looking for such a mutation poolside at our kids’ swim meet to my neighbor, good friend, and colleague Pete Zimmerman, a human geneticist working as a post-doc at the time with Tom Nutman in the Laboratory of Parasitic Diseases of NIAID and now a Professor at Case Western Reserve University School of Medicine. Pete agreed to collaborate, and using a heteroduplex DNA mobility shift assay for polymorphism detection he found among 100 blood donors from the NIH Clinical Center Blood Bank, 21 individuals with a massive shift: 20 heterozygotes and one homozygote for what was eventually named *CCR5Δ32*, which we later nicknamed “the mother of all mutations in the molecule of the year.” All of our criteria had been met: it was common (but restricted mainly to Caucasians), and the 32 base pair deletion caused a massive truncation incompatible with expression and function. Next we received approval from NIAID’s Division of AIDS to analyze several thousand DNA samples from participants in the Multicenter AIDS Cohort Study (MACS), and we collaborated with HIV/AIDS expert Tony Fauci, the Director of NIAID, whose laboratory was right around the corner from mine, to obtain DNA from two cohorts of long-term non-progressors and one group of HIV exposed-uninfected (EU) individuals. As predicted, compared to the frequency in the general population, *CCR5Δ32* homozygosity was markedly increased by about fivefold in the EU population. However, our analysis of the critical MACS samples was delayed by 2 months during which Steven O’Brien from the NCI of NIH, who had custody of the MACS samples and had been directed to send them to us as well as to Rick Koup at NYU for analysis, conducted his own study of *CCR5Δ32* in HIV. We finally received the samples a few weeks before his paper was published in *Science*, and completed our study validating the second and third parts of our hypothesis that homozygotes should be underrepresented from the HIV-infected population and that heterozygotes would have a delayed time from infection to the diagnosis of AIDS. Importantly, homozygotes in the general population appeared to be healthy. Together, our paper published in *Molecular Medicine*, O’Brien’s *Science* paper, and papers reporting independent discoveries of *CCR5Δ32* by the groups of Marc Parmentier in Brussels in *Nature*, and Rick Koup at the Aaron Diamond AIDS Research Center in *Cell* provided strong proof of principle for targeting CCR5 in the treatment of patients with HIV/AIDS (10, 12). Thirteen years later, this discovery culminated in FDA approval of the small molecule CCR5 antagonist Selzentry (maraviroc, from Pfizer) for the treatment of CCR5-tropic HIV. In addition, the “Berlin patient,” an HIV^+^ individual who developed leukemia and was functionally cured of HIV by a transplant with bone marrow from a *CCR5Δ32* homozygote given after leukemia chemotherapy, provided proof-of-principle that targeting CCR5 might be a cure strategy in HIV/AIDS. The hope for the future is that cure strategies will be available for every HIV^+^ individual through deliberate genome editing of CCR5.

Overall, we were pleased that our NIAID collaborative group contributed the four main arms for the underlying proof-of-principle discoveries about CCR5 and HIV: the independent cloning of CCR5, demonstration that CCR5 was an M-tropic HIV coreceptor, discovery of *CCR5Δ32*, and demonstration that *CCR5Δ32* is an HIV genetic restriction factor at the population level. The foundational discoveries on which the CCR5 work rested also came from NIH: Paolo’s discovery at the NCI of HIV suppressive activity for CC chemokines (6) and Ed’s discovery at NIAID of fusin/CXCR4 as the first HIV coreceptor (7). After this work, my lab went on to discover the first beneficial role for CCR5 as a host defense factor in West Nile virus infection (13, 14).

## Conflict of Interest Statement

The author declares that the research was conducted in the absence of any commercial or financial relationships that could be construed as a potential conflict of interest.
